# Functioning, temporal resolution, and functional health literacy in children aged nine to twelve years

**DOI:** 10.1590/2317-1782/e20240320en

**Published:** 2026-03-31

**Authors:** Jaqueline Batista Diniz Gonçalves, Andrezza Gonzalez Escarce, Stela Maris Aguiar Lemos

**Affiliations:** 1 Programa de Pós-graduação em Ciências Fonoaudiológicas, Universidade Federal de Minas Gerais – UFMG - Belo Horizonte (MG), Brasil.; 2 Programa de Pós-graduação em Ciências Fonoaudiológicas, Departamento de Fonoaudiologia, Faculdade de Medicina, Universidade Federal de Minas Gerais – UFMG - Belo Horizonte (MG), Brasil.

**Keywords:** Students, Health Literacy, Hearing, Interdisciplinary Communication, Communication Barriers, Information Literacy

## Abstract

**Purpose:**

To verify the association between functioning, auditory temporal resolution, functional health literacy, and sociodemographic data of children aged 9 to 12 years.

**Methods:**

This is an analytical, cross-sectional, observational study with a non-probabilistic sample of 53 schoolchildren of both sexes, aged 9 to 12 years, and their caregivers. The following procedures were performed: medical history survey, application of the CCEB questionnaire, Token Test, auditory evaluation, tympanometry, evaluation of auditory temporal resolution using the Random Gap Detection Test, Functional Health Literacy Scale, and coding of category d350 of the International Classification of Functioning, Disability and Health (ICF). The study performed descriptive and bivariate analyses.

**Results:**

The descriptive analysis showed that most participants were adequate regarding category d350 and functional health literacy. The bivariate analysis indicated statistical significance between the ICF category – conversation d350 and functional health literacy (p=0.02).

**Conclusion:**

Communication performance may have influenced the functional health literacy of schoolchildren, making it essential to study the intervening factors among these variables.

## INTRODUCTION

Temporal auditory skills are the central auditory processing skills responsible for analyzing and interpreting sound events to understand auditory information^(^1,2^)^. Thus, they process acoustic signals as a function of reception time to detect rapid changes in sound stimuli and are related to numerous stages of everyday listening, favoring, among other attributions, the decoding of the spoken message^(^1,3^)^.

Changes in temporal resolution can result in difficulties in identifying small acoustic variations in speech, thus hindering interpretation of the heard message and decoding of the written message^(^1^)^. Understanding verbal messages correctly helps people comprehend, interpret, and apply information and make assertive decisions regarding their health^(^4^)^.

A person's ability to understand, interpret, and apply health information directed to them enables adequate functional health literacy^(^5^)^. In short, the latter refers to literacy and implies knowledge, motivation, and competence of people to access, understand, evaluate, and apply health information to judge and make daily decisions about healthcare, disease prevention, and health promotion to maintain or improve quality of life^(^6^)^. It provides the person with the right to information about self-care and health services^(^5,7^)^.

Low functional health literacy can hinder health promotion and education. It is also associated with risky behaviors, such as reduced self-care, increased hospitalizations, and costs^(^8^)^. In addition to the ability to read, write, and understand verbal messages, the ability to make decisions based on the health information obtained is rather important^(^9^)^. A person with a satisfactory level of functional health literacy navigates health environments more easily, thus having better conditions for self-care and for managing health conditions^(^10,11^)^.

When it comes to children, adults must guide and direct them to promote understanding and self-care in their health needs^(^12^)^. Such actions can improve children’s health conditions, potentially reducing the use of more complex procedures in health services^(^12,13^)^. Furthermore, parental literacy is of paramount importance for child development, as family attitudes and customs influence the management of care and self-care in the health of family members^(^11,12^)^.

Validated scales are necessary to describe and measure aspects of health, functioning, and disability of children and their families. These include the International Classification of Functioning, Disability and Health (ICF)^(^13^)^, recognized by the World Health Organization (WHO) as an international standard for individual and population scale, and accepted as one of the United Nations’ social classifications^(^14^)^.

Using the ICF conceptual basis makes it possible to characterize speech aspects and the social consequences of their impairments in children's lives^(^15^)^. ICF use and analysis have enabled a greater understanding of needs, including those related to impaired functioning, its severity, and the impact of interventions, as well as verifying which environmental factors may be associated^(^16^)^.

Considering the possible relationship of verbal message interpretation and comprehension with health-friendly decision-making, this study aimed to verify the association between functioning, auditory temporal resolution, functional health literacy, and sociodemographic data of children aged 9 to 12 years.

## METHODS

This is a preliminary analytical, observational, cross-sectional study with a non-probabilistic sample of 53 schoolchildren of both sexes, aged 9 to 12 years, and their caregivers (parents or guardians). The study was approved by the institution’s Research Ethics Committee under approval number 2.093.022. All participants signed an informed consent form and an informed assent form, in accordance with the recommendations of Resolution 466 of the Brazilian National Health Council.

The research setting was the Functional Speech-Language-Hearing (SLH) Health Observatory at the Medical School of the Federal University of Minas Gerais, in Belo Horizonte, Brazil. Participants attended previously scheduled appointments with the researcher.

The research was publicized on social media and in the SLH clinic of the Clinics Hospital of Belo Horizonte to recruit participants. The researcher contacted those interested in participating by telephone and then scheduled them for the necessary assessments and procedures.

The study inclusion criteria were children aged 9 to 12 years; who signed an informed assent form; whose parents agreed to and signed an informed consent form; who performed adequately in cognitive aspects as determined by screening results of the Token Test^(^17^)^; and who had adequate auditory acuity, identified by tonal, vocal, and tympanometry auditory assessment.

One of the researchers administered the instruments in a private room. Responses were recorded on questionnaires and assessment protocols. The following procedures were followed for the children's assessment:

- Medical history survey: developed by the researchers for application with parents/guardians, with responses transcribed in real time during the interview. The instrument has seven items with the following sections: individual characteristics of the children, sociodemographic questions, health history, and information related to temporal auditory skills. The items were:

1. Identification.
2. Why did you seek SLH care for your child? () the doctor requested it; () the school requested it; () I noticed SLH-related impairment(s); () other
3. Is your child undergoing any treatment? ( ) yes ( ) no
4. How would you rate your child's health? () Good () Neither good nor poor () Poor
5. Do you think your child hears well? ( ) yes ( ) no
6. How would you rate your child's hearing? ( ) Good ( ) Poor
7. Does your child have difficulty with any of the issues below?
  a) Paying attention for longer. ( ) yes ( ) no
  b) Talking in noisy environments. ( ) yes ( ) no
  c) Talking to several people at the same time. ( ) yes ( ) no
  d) Doing school activities. ( ) yes ( ) no
  e) Following instructions. ( ) yes ( ) no

- Token Test – Reduced Version^(^17^)^: The Token Test, reduced version^(^17^)^, is used for screening initial cognitive aspects. It was developed to identify subtle changes in verbal comprehension and assess the integrity of the cognitive components of language. The application is based on verbal commands for the participant to execute, measuring linguistic development in relation to age and schooling. The test application uses geometric pieces of different colors and sizes, facilitating the execution of the instructions and making the protocol more accessible to children. The test has six parts. Part 1 has seven items; Parts 2, 3, 4, and 5 have four items each; and Part 6 has 13 items. Parts 1, 3, and 5 use all geometric pieces (large and small), while Parts 2, 4, and 6 use only the large pieces; the small pieces are kept covered during the application.
- Brazilian Economic Classification Criteria (CCEB)^(^18^)^: Developed by the Brazilian Association of Market Research (ABEP). Its objective is to estimate the purchasing power of families through household characteristics and goods, and the householder’s education level. It is applied in interviews, and the questions refer to the number of items available in the residence, the existence of public services, and the householder’s education level. For its analysis, each item has a specific score, whose sum determines the corresponding classification into six possible class categories: A, B1, B2, C1, C2, D-E. The greater the number of items and the higher the householder’s education level, the higher the score, with class A being the highest level.
- Hearing Assessment^(^19^)^: The assessment was conducted in an acoustically treated room, ensuring appropriate conditions for the application of audiological procedures. Initially, the external auditory canal (EAC) was inspected to verify the absence of obstructions or conditions that could interfere with subsequent stages of the assessment. Next, acoustic immittance analysis was performed, consisting of tympanometry and the testing of contralateral and ipsilateral acoustic reflexes using a Madsen Astera^(^2^)^ tympanometer with a 226-Hz probe. Finally, a conventional audiological assessment was performed, consisting of pure-tone audiometry to determine hearing thresholds and speech audiometry to assess speech detection and recognition ability. Results were considered within normal limits if they had a type A tympanogram, bilateral acoustic reflexes, hearing thresholds equal to or better than 15 dB HL in both ears, and compatible responses in audiometric screening with sweep technique at 500 to 4000 Hz, according to criteria established by the specialized literature^(^19^)^.
- Temporal resolution was assessed using the Random Gap Detection Test (RGDT)^(^20^)^, which measures the ability to detect silence intervals between two sound stimuli. This diotic test consists of presenting pairs of pure tones at 500, 1000, 2000, and 4000 Hz, with time intervals randomly varying between 0, 2, 5, 10, 15, 20, 25, 30, and 40 milliseconds. Participants were instructed to verbally respond whether they perceived one or two sounds. Results with an average detection time of less than or equal to 10 milliseconds were considered within the normal range. It was applied in an acoustically treated room, ensuring controlled evaluation conditions.
- The researchers developed the Functional Health Literacy Scale for children to evaluate their functional health literacy, considering the concept of functional health literacy, the age range of the study, and children aged 9 years or older, who can already make decisions^(^4-6^)^. The instrument has eight questions, in which the child chooses one of three response options: always, sometimes, or never. An illustrated visual scale was used, as presented in Appendix A, to help them understand the alternatives, especially younger groups. It was applied individually, with assisted reading when necessary, respecting the child's pace and comprehension level.
- The ICF^(^13^)^ is a tool developed by the WHO to systematize and describe aspects related to people’s functioning and health conditions. This classification organizes, in an integrated way, different domains of a person's life in the face of a specific health condition. This study considered the conversation category (d350)^(^13^)^, which includes performance in conversation, initiating, maintaining, and ending an exchange of thoughts and ideas, carried out through written, oral, sign language, or other forms of language, with one or more known or unknown people, in formal or informal settings^(^13^)^. Only the qualifiers 0 (no difficulty) and 8 (unspecified difficulty) were used. It is worth noting that qualifier 8 is used when there is a difficulty, but its degree of difficulty cannot be determined. Qualifiers were selected based on interview transcripts and ICF questionnaire answers given by the caregivers^(^13^)^. The scores assigned to the responses (0 = Never, 1 = Sometimes, and 2 = Always) were summed to assess whether health literacy was considered adequate or inadequate. The median (6.0 points) was used as a cutoff point to classify the participants according to their level of literacy.

### Data analysis

This study defined the category conversation (d350)^(^13^)^ as the dependent variable. The independent variables (sex, education, type of school, and economic classification) were sociodemographic variables, functional health literacy, and temporal skills.

Descriptive analysis was performed using the frequency distribution of categorical variables and analysis of measures of central tendency and dispersion of continuous variables. Pearson's chi-square test was used for association analyses, adopting a 5% significance level (p < 0.05).

The responses to the instruments were organized and digitized in a specific database. The SPSS software, version 25.0, was used for data entry, processing, and analysis.

## RESULTS

Most of the 53 participating students were 9 years old (30.8%), with a mean age of 10.31 years, a standard deviation of 1.09, and a median of 10 years. The majority were male (58.5%), attended public school (77.4%), belonged to CCEB’s A/B class (51.9%), had no impairment in the conversation category - d350 (52.8%), and had abnormal RGDT results (79.2%). Moreover, most were in the 5^th^ grade (28.3%) and had an inadequate result (62.3%) on the Functional Health Literacy Scale (Table 1).

**Table 1:** Characteristics of the students evaluated

	N	%
**Sex**		
Females	22	41.5
Males	31	58.5
Total	53	100.0
**Age**		
9 years	16	30.2
10 years	13	24.5
11 years	14	26.4
12 years	10	18.9
Total	53	100.0
**Grade in school**		
3^rd^ grade	2	3.8
4^th^ grade	13	24.5
5^th^ grade	15	28.3
6^th^ grade	14	26.4
7^th^ grade	9	17.0
Total	53	100.0
**School type**		
Public	41	77.4
Private	12	22.6
Total	53	100.0
**CCEB**		
A/B	28	52.8
C-D/E	25	47.2
Total	53	100
**CIF d350**		
No difficulty	28	52.8
Unspecified difficulty	25	47.2
**Literacy**		
Adequate	20	37.7
Inadequate	33	62.3
Total	53	100
**RGDT**		
Adequate	11	20.8
Inadequate	42	79.2
Total	53	100.0


**Caption:** CCEB = Brazilian Economic Classification Criteria; ICF = International Classification of Functioning; RGDT = Random Gap Detection Test

The analysis of the Functional Health Literacy Scale by question found that most participants answered "always" in questions Q3 (49.1%), Q4 (54.7%), and Q8 (64.2%); most respondents answered “sometimes” in Q1 (54.7%), Q2 (39.6%), and Q7 (43.4%); and most respondents answered “never” in Q5 (41.5%) (Table 2).

**Table 2:** Analysis of the Functional Health Literacy Scale

	N	%
**Q1: When you go to a speech-language-hearing pathologist, doctor, dentist, or other healthcare professional, do you usually pay attention to the instructions they give to your parents?**		
Always	22	41.5
Sometimes	29	54.7
Never	2	3.8
Total	53	100.0
**Q2: When you go to the doctor, speech-language-hearing pathologist, dentist, or other healthcare professional, do you usually ask questions about your health?**		
Always	12	22.6
Sometimes	21	39.6
Never	20	37.7
Total	53	100
**Q3: Can you understand the instructions speech-language-hearing pathologists, doctors, dentists, or other healthcare professionals give to you?**		
Always	26	49.1
Sometimes	18	34.0
Never	9	17.0
Total	53	100.0
**Q4: Can you understand the explanations your parents give about your health?**		
Always	29	54.7
Sometimes	17	32.1
Never	7	13.2
Total	53	100.0
**Q5: Do you usually ask your parents about the treatment you are receiving from the speech-language-hearing pathologist?**		
Always	14	26.2
Sometimes	17	32.1
Never	22	41.5
Total	53	100.0
**Q6: Do you usually ask your parents about the treatment/appointment you are having with the doctor?**		
Always	17	32.1
Sometimes	18	34.0
Never	18	34.0
Total	53	100.0
**Q7: Do you usually ask your parents about the treatment/consultation you are receiving from other healthcare professionals?**		
Always	14	26.4
Sometimes	23	43.4
Never	16	30.2
Total	53	100.0
**Q8: Do you usually pay attention to the guidance given by speech-language-hearing pathologists, doctors, and dentists?**		
Always	34	64.2
Sometimes	13	34.5
Never	6	11.3
Total	53	100.0


**Caption:** Q = question

An association analysis was performed between the ICF d350 category and the selected study variables using Pearson's chi-square test, finding it was statistically significantly associated with functional health literacy (p = 0.012). Children without communication complaints had adequate levels of functional health literacy, while those with communication complaints had a higher frequency of difficulties in this aspect (Table 3).

**Table 3:** Association analysis between ICF d350 and selected variables

**Variables**	ICF d350		p-value*
	Unspecified difficulty	No difficulty	
	**N(%)**	**N(%)**	
**Sex**			0.442
Females	9 (36.0)	13 (46.4)	
Males	16 (64.0)	15 (53.6)	
Total	25 (100.0)	28 (100.0)	
**Age**			0.948
9 years	7 (28.7)	9 (33.3)	
10 years	6 (24.0)	7 (25.9)	
11 years	7 (28.0)	7 (25.9)	
12 years	5 (20.0)	4 (14.8)	
Total	25 (100.0)	27 (100.0)	
**Grade in school**			0.518
3^rd^ grade	2 (8.0)	0 (0.0)	
4^th^ grade	5 (20.0)	8 (28.6)	
5^th^ grade	6 (24.0)	9 (32.1)	
6^th^ grade	7 (28.0)	7 (25.0)	
7^th^ grade	5 (20.0)	4 (14.3)	
Total	25 (100.0)	28 (100.0)	
**School type**			0.823
Public	19 (76.0)	22 (78.6)	
Private	6 (24.0)	6 (21.4)	
Total	25 (100.0)	28 (100.0)	
**CCEB**			0.797
A/B	12 (50.0)	15 (53.6)	
C-D/E	12 (50.0)	13 (46.4)	
Total	24 (100.0)	28 (100.0)	
**RGDT**			0.898
Adequate	5 (20.0)	6 (21.4)	
Inadequate	20 (80.0)	22 (72.6)	
Total	25 (100.0)	28 (100.0)	
**Health literacy**			0.012*
Adequate	5 (20.0)	15 (53.5)	
Inadequate	20 (80.0)	16.4 (24.5)	
Total	25 (100.0)	28 (100.0)	

Pearson's chi-square test

* p-value ≤ 0.05

**Caption:** CCEB = Brazilian Economic Classification Criteria; ICF = International Classification of Functioning, Disability and Health; RGDT = Random Gap Detection Test

The association analysis between ICF d350 and the Functional Health Literacy Scale, by question, found it statistically significantly associated with Q1 (p = 0.040) and Q2 (p = 0.035), with a tendency for those classified as yes in the ICF code to answer never in the functional literacy questions. The other questions had no statistically significant results (Table 4).

**Table 4:** Association analysis between ICF d350 and questions from the Functional Health Literacy Scale

**Variables**	**ICF d350**		**p-value** *
	**Unspecified difficulty**	**No difficulty**	
	**N (%)**	**N (%)**	
**Q1: When you go to a speech-language-hearing pathologist, doctor, dentist, or other healthcare professional, do you usually pay attention to the instructions they give to your parents?**			0.040
Always	7 (28.0)	15 (53.6)	
Sometimes	18 (72.0)	11 (39.3)	
Never	0 (0.0)	2 (7.1)	
Total	25 (100.0)	28 (100.0)	
**Q2: When you go to the doctor, speech-language-hearing pathologist, dentist, or other healthcare professional, do you usually ask questions about your health?**			0.035
Always	4 (16.0)	8 (28.6)	
Sometimes	7 (28.0)	14 (50.0)	
Never	14 (56.0)	6 (21.4)	
Total	25 (100.0)	28 (100.0)	
**Q3: Can you understand the instructions speech-language-hearing pathologists, doctors, dentists, or other healthcare professionals give to you?**			0.659
Always	13 (52.0)	13 (46.4)	
Sometimes	9 (36.0)	9 (32.2)	
Never	3 (12.0)	6 (21.4)	
Total	25 (100.0)	28 (100.0)	
**Q4: Can you understand the explanations your parents give about your health?**			0.504
Always	12 (48.0)	17 (60.7)	
Sometimes	10 (40.0)	7 (25.0)	
Never	3 (12.0)	4 (14.3)	
Total	25 (100.0)	28 (100.0)	
**Q5: Do you usually ask your parents about the treatment you are receiving from the speech-language-hearing pathologist?**			0.202
Always	4 (16.0)	10 (35.8)	
Sometimes	8 (32.0)	9 (32.1)	
Never	13 (52.0)	9 (32.1)	
Total	25 (100.0)	28 (100.0)	
**Q6: Do you usually ask your parents about the treatment/appointment you are having with the doctor?**			0.668
Always	7 (28.0)	10 (35.7)	
Sometimes	10 (40.0)	8 (28.6)	
Never	8 (32.0)	10 (35.7)	
Total	25 (100.0)	28 (100.0)	
**Q7: Do you usually ask your parents about the treatment/consultation you are receiving from other healthcare professionals?**			0.289
Always	7 (28.0)	7 (25.0)	
Sometimes	13(52.0)	10 (35.7)	
Never	5 (20.0)	11 (39.3)	
Total			
**Q8: Do you usually pay attention to the guidance given by speech-language-hearing pathologists, doctors, and dentists?**			0.161
Always	13 (52.0)	21 (75.0)	
Sometimes	9 (36.0)	4 (14.3)	
Never	3 (12.0)	3(10.7)	
Total	25 (100.0)	28 (100.0)	

* Pearson's chi-square Test

**Caption:** Q = question

## DISCUSSION

This study analyzed the relationship between functioning, temporal skills, health literacy, and sociodemographic aspects of schoolchildren aged 9 to 12 years. The sample distribution showed that most children were male, in the 5^th^ grade, and attended public school.

Regarding the CCEB, most came from the A/B economic class and attended regular school, which may have contributed to the positive results in functional health literacy performance. A previous study points out that functional health literacy is related to social, school, and quality of life conditions^(^21^)^. Furthermore, the level of functional health literacy may vary according to sample characteristics, such as sociodemographic, cultural, and family issues^(^22^)^.

Using ICF’s biopsychosocial approach to characterize functional aspects related to conversational performance allows us to describe possible factors related to functioning, disability, and the context of the participants, which may influence the acquisition of skills important for social participation, such as functional health literacy^(^16^)^. The selection of qualifiers in this study, through interview transcripts and ICF questionnaire answers by caregivers, allowed us to identify the family's perception of their children’s conversational skills.

The evidence that most children evaluated had no complaints associated with the conversation category (d350) is important in this study. Specifically, few difficulties were reported related to situations such as conversing in noisy environments, interacting with multiple interlocutors simultaneously, maintaining attention for extended periods, and following verbal instructions. These aspects are directly related to the functional demands of the child's school, family, and social routine.

On the other hand, children with communication complaints performed consistently lower on the Functional Health Literacy Scale, suggesting a possible association between functional limitations in communication and a lower ability to understand and use health-related information. This finding reinforces the hypothesis that difficulties in the communication domain can directly impact how children access, process, and respond to guidance in clinical, school, and family contexts. This indicates an association between functional health literacy and an adequate ability to understand messages, as this ability is necessary to analyze and understand health information, which favors adequate functional health literacy^(^16^)^.

These results are consistent with a previous study that points to communication as a central skill for promoting health literacy from childhood^(^23^)^, considering that understanding, interpreting, and applying information depend on well-developed linguistic and cognitive skills. Furthermore, the communicative environment, ​​such as noise, number of interlocutors, and complexity of instructions, can be an important modulating factor of these skills, especially among children in the language acquisition and consolidation phase^(^23^)^.

Most students analyzed had inadequate results in the Functional Health Literacy Scale, applied to the study population. Data analysis of previous studies with adolescents and adults^(^21,22^)^ revealed that most of them had adequate functional health literacy scores, using various instruments.

More than a third of the participants performed inadequately in functional health literacy, a result that deserves attention, especially since it involves a population in the developmental phase. The literature has demonstrated a significant association between higher levels of education and greater competence in functional health literacy^(^5,7,21,22^)^. Studies indicate that individuals tend to be less likely to have difficulties in this skill, since schooling is directly related to vocabulary acquisition, comprehension ability, and the functional use of language in everyday life^(^7,24^)^. The sample in this study consisted of children in formal education, learning to read and write, enrolled in elementary school. Hence, it is plausible to assume that their performance in functional health literacy is related to their stage of cognitive and linguistic development. The ability to understand, interpret, and apply health information is still under construction, being influenced by both school factors and the family and social environment.

The analysis of the RGDT test showed that most children had abnormal results. A study with children with phonological disorders found poor performance in all temporal processing tests used in research subjects^(^25^)^. In this study, most children reported complaints about school difficulties, which may be correlated with performance on the RGDT test. Furthermore, a possible explanation for the high number of abnormal results is that parents with greater concern for their children's development responded to the invitation made on social media.

The association analysis between the RGDT test result and the conversation category (d350) found no statistical significance. As this category was answered by applying the ICF questionnaire to parents/guardians and surveying medical history, most reported that the children did not present difficulties in this category’s conversational skills. This result should be interpreted with caution. In another study, participants reported difficulty communicating in family contexts, such as talking at home or following instructions in environments with multiple stimuli^(^15^)^. Moreover, the children reported talking little with their parents, with few moments of conversation in the family environment^(^15^)^. Such discrepancies may be associated with different sources of information (child's self-report vs. caregiver's report), the subjectivity of parental perception, or contextual characteristics that influence the manifestation of communication difficulties in specific situations. Therefore, further investigations of this skill are needed with specific assessments of students in subsequent studies and greater clarification regarding family dynamics.

The possible relationship between temporal aspects and the conversation category (d350) makes it possible to identify limitations in reception tasks and the expression of oral language, which can influence social participation activities. Besides these factors, personal and environmental factors must be considered, including school age and learning difficulties, to understand each child’s particularities^(^26,27^)^. Thus, using qualifiers in association with personal skills favors the visualization of activity limitations and participation restrictions that may be caused by impairments in auditory functions associated with environmental factors, and such impairments may influence people's quality of life^(^27,28^)^.

As limitations of the present study, it is worth highlighting the sample size and selection (non-probabilistic), which do not allow the generalization of the results. In addition, despite the wide dissemination for recruitment using social media, adherence was low for the research. Furthermore, the reduced number of sample participants from private schools compromises the analysis of the school type variable.

There are advances that deserve to be considered, such as the triangulation between the biopsychosocial model, temporal resolution, and health literacy. It is also an innovative study in addressing health literacy and auditory processing in schoolchildren, which considers the biopsychosocial health model advocated by the WHO, integrating biological, psychological, and social aspects to understand the individual. The methodological proposal contributes to bringing clinical practice closer to international guidelines for assessment focused on functioning, promoting a broader and more contextualized view of child development. From a practical point of view, the findings reinforce the importance of considering health literacy from childhood, especially in populations in the language acquisition phase and in school contexts. Detecting communication difficulties and problems understanding health information can lead to more effective interdisciplinary interventions, with positive effects on academic performance, adherence to clinical guidelines, and development of communicative autonomy.

It is recommended that future studies expand the sample, include multiple informants (child, parents, and teachers), and apply standardized and validated instruments for different linguistic and cultural contexts. Continued research that articulates functional aspects, auditory skills, and health literacy may contribute to the design of more effective educational and preventive strategies, focusing on promoting children's health and strengthening communication as a determinant of care.

## CONCLUSION

The results revealed an association between schoolchildren’s conversation category (d350) and functional health literacy. Guided by the principles of comprehensive care and health promotion and using the conceptual basis of the ICF, it is possible to associate performance in functional health literacy and conversational skills with children’s Activities, Participation, and Environmental Factors.

The study showed that functional health literacy is composed of complex and multifactorial social structures. This reinforces the importance of further studies that associate personal, environmental, and social skills to better understand functional health literacy in children.

## Appendix A Functional Health Literacy Scale

**Table:**

**HEALTH LITERACY IN CHILDREN AND ADOLESCENTS**											
**1.**	When you go to a speech-language-hearing pathologist, doctor, dentist, or other healthcare professional, do you usually pay attention to the instructions they give to your parents?										
		
	Always		Sometimes					Never			
**2.**	When you go to the doctor, speech-language-hearing pathologist, dentist, or other healthcare professional, do you usually ask questions about your health?										
		
	Always				Sometimes			Never			
**3.**	Can you understand the instructions speech-language-hearing pathologists, doctors, dentists, or other healthcare professionals give to you?										
		
	Always			Sometimes		Never					
**4.**	Can you understand the explanations your parents give about your health?										
		
	Always	Sometimes					Never				
**5.**	Do you usually ask your parents about the treatment you are receiving from the speech-language-hearing pathologist?										
		
	Always			Sometimes		Never					
**6.**	Do you usually ask your parents about the treatment/appointment you are having with the doctor?										
		
	Always	Sometimes							Never		
**7.**	Do you usually ask your parents about the treatment/consultation you are receiving from other healthcare professionals?										
		
	Always	Sometimes								Never	
**8.**	Do you usually pay attention to the guidance given by speech-language-hearing pathologists, doctors, and dentists?										
		
	Always			Sometimes							Never
