# Supplementary material for: Functioning, temporal resolution, and functional health literacy in children aged nine to twelve years
Source: Codas. 2026 Mar 31;38(3):e20240320. doi: 10.1590/2317-1782/e20240320en (PMC13142684; doi:10.1590/2317-1782/e20240320en)
Supplement: Translation

# Translation: Funcionalidade, resolução temporal e letramento funcional em saúde em crianças de nove a doze anos

**DOI:** 10.1590/2317-1782/e20240320pt

**Authors:** Jaqueline Batista Diniz Gonçalves, Andrezza Gonzalez Escarce, Stela Maris Aguiar Lemos

**Affiliations:** 1 Programa de Pós-graduação em Ciências Fonoaudiológicas, Universidade Federal de Minas Gerais – UFMG - Belo Horizonte (MG), Brasil.; 2 Programa de Pós-graduação em Ciências Fonoaudiológicas, Departamento de Fonoaudiologia, Faculdade de Medicina, Universidade Federal de Minas Gerais – UFMG - Belo Horizonte (MG), Brasil.

**Keywords:** Estudantes, Letramento em Saúde, Audição, Comunicação Interdisciplinar, Barreiras de Comunicação, Competência em Informação

## Abstract

**Objetivo:**

Verificar a associação entre a funcionalidade, a habilidade auditiva de resolução temporal, letramento funcional em saúde e dados sociodemográficos de crianças entre nove e doze anos de idade.

**Método:**

Trata-se de estudo observacional analítico de recorte transversal com amostra não probabilística composta por 53 escolares, de ambos os sexos, com idade entre nove e 12 anos e seus acompanhantes. Foram realizados os seguintes procedimentos: anamnese, aplicação do questionário CCEB, Teste Token, avaliação auditiva, imitanciometria, avaliação da habilidade auditiva de resolução temporal mediante aplicação do Random Gap Detection Test, Escala de Letramento Funcional em Saúde, codificação da categoria d350 da Classificação internacional de Funcionalidade (CIF). Foram realizadas análises descritiva e bivariada.

**Resultados:**

Na análise descritiva foi observado que a maioria dos participantes apresentou adequação da categoria d350 e do Letramento Funcional em saúde. A análise bivariada indicou significância estatística entre a categoria da CIF - comunicação d350 e Letramento Funcional em Saúde (p=0,02).

**Conclusão:**

O desempenho na comunicação pode ter influenciado o letramento funcional em saúde dos escolares, de modo que se torna essencial estudar os fatores intervenientes entre estas variáveis.

## INTRODUÇÃO

As habilidades auditivas temporais, dentre as habilidades do Processamento Auditivo Central, são responsáveis por analisar e interpretar os eventos sonoros para possibilitar a compreensão da informação auditiva^(^1,2^)^. Sendo assim, são responsáveis pelo processamento do sinal acústico em função do tempo de recepção, por detectar mudanças rápidas no estímulo sonoro e se relacionam com inúmeras etapas da escuta cotidiana, que favorece, dentre outras atribuições, a decodificação da mensagem falada^(^1,3^)^

Alterações na resolução temporal podem resultar em dificuldades para identificar pequenas variações acústicas na fala e, consequentemente, dificuldades em interpretar a mensagem ouvida e na decodificação da mensagem escrita^(^1^)^. A correta compreensão da mensagem verbal favorece às pessoas entenderem, interpretarem e aplicarem informações e tomarem decisões assertivas quanto à sua saúde^(^4^)^.

No âmbito da saúde, a capacidade da pessoa para entender, interpretar e aplicar as informações que lhe são direcionadas possibilitam o adequado Letramento Funcional em Saúde^(^5^)^. Em suma, o Letramento Funcional em Saúde se refere à alfabetização e implica conhecimento, motivação e competência das pessoas para acessar, compreender, avaliar e aplicar informações de saúde para julgar e tomar decisões no cotidiano sobre cuidados de saúde, prevenção de doenças e promoção da saúde para manter ou melhorar a qualidade de vida^(^6^)^. Ele propicia à pessoa o direito à informação sobre o autocuidado e os serviços de saúde^(^5,7^)^.

Um baixo Letramento Funcional em Saúde pode dificultar a promoção e a educação em saúde. Além disso, está associado aos comportamentos de risco, tais como redução do autocuidado, aumento de hospitalizações e custos^(^8^)^. Considera-se importante, além da capacidade de leitura, escrita e compreensão da mensagem verbal, a capacidade que o sujeito possui para tomar decisões diante das informações de saúde obtidas^(^9^)^. Uma pessoa com nível de Letramento Funcional em Saúde satisfatório transita melhor pelos ambientes de saúde, tendo, assim, melhores condições para o autocuidado e para o gerenciamento de condições de saúde^(^10,11^)^.

Em se tratando de crianças, vale destacar a importância do adulto em orientar e direcioná-las com o intuito de favorecer a compreensão e o autocuidado em demandas de sua saúde^(^12^)^. Tais ações podem possibilitar melhoras das condições de saúde do indivíduo desde a infância, o que poderá contribuir na redução da utilização de procedimentos mais complexos nos serviços de saúde^(^12,13^)^. Além disso, é necessário considerar que o letramento parental é de suma importância para o desenvolvimento da criança, pois as atitudes e os costumes familiares influenciam na gestão do cuidado e autocuidado em saúde dos membros da família^(^11,12^)^.

Para descrever e mensurar os aspectos de saúde, a funcionalidade e a incapacidade das crianças e seus familiares é necessário o uso de escalas validadas. Dentre elas, cita-se a Classificação Internacional de Funcionalidade, Incapacidade e Saúde (CIF)^(^13^)^, reconhecida pela Organização Mundial de Saúde (OMS) como padrão internacional em escala individual e populacional e aceita como uma das classificações sociais das Nações Unidas^(^14^)^.

A utilização da base conceitual da CIF possibilita caracterizar os aspectos de fala e as repercussões sociais de suas alterações na vida das crianças^(^15^)^. O uso e a análise da CIF têm possibilitado um maior conhecimento de demandas, incluindo as de alterações na funcionalidade, sua gravidade e o impacto das intervenções, além da verificação de quais fatores ambientais podem estar associados^(^16^)^.

Tendo em vista a possível relação entre interpretação e compreensão da mensagem verbal com tomadas de decisões favoráveis à saúde, o presente estudo teve como objetivo verificar a associação entre funcionalidade, habilidade auditiva de resolução temporal, letramento funcional em saúde e dados sociodemográficos de crianças entre nove e doze anos de idade.

## MÉTODO

Trata-se de estudo preliminar com delineamento observacional analítico, de recorte transversal, com amostra não probabilística composta por 53 escolares, de ambos os sexos, com idade entre nove e 12 anos e seus acompanhantes (pais ou responsáveis). O estudo foi aprovado pelo Comitê de Ética em Pesquisa da instituição, sob o parecer número 2.093.022. Todos os participantes assinaram o termo de consentimento livre e esclarecido e o termo de assentimento livre e esclarecido, de acordo com as recomendações da resolução do CNS 466.

O cenário de pesquisa foi o Observatório de Saúde Funcional em Fonoaudiologia da Faculdade de Medicina da Universidade Federal de Minas Gerais, em Belo Horizonte, mediante agendamento prévio, realizado pela pesquisadora.

Para recrutamento dos participantes foi realizada divulgação da pesquisa em mídias sociais e nos ambulatórios de fonoaudiologia do Hospital das Clínicas de Belo Horizonte, MG. Os interessados em participar foram contatados mediante contato telefônico pela pesquisadora e, em seguida, realizado agendamento para realização das avaliações e procedimentos necessários.

Foram definidos como critérios de inclusão para o estudo: crianças com idade entre nove e 12 anos, cujos pais concordaram e assinaram o Termo de Consentimento Livre e Esclarecido (TCLE), assim como as crianças, o Termo de Assentimento Livre e Esclarecido (TALE); adequado desempenho dos aspectos cognitivos mediante resultado de triagem, realizada por meio da aplicação do Token Test^(^17^)^; e adequada acuidade auditiva identificada pela avaliação auditiva tonal, vocal e imitanciometria.

A aplicação dos instrumentos foi realizada por uma das pesquisadoras, em sala reservada. As respostas foram registradas nos questionários e protocolos de avaliação. Para a avaliação das crianças foram realizados os seguintes procedimentos:

- Anamnese: elaborada pelas pesquisadoras, para aplicação com os pais/responsáveis, sendo as respostas transcritas em tempo real durante a entrevista. O instrumento é constituído por sete itens, que contêm os eixos: características individuais das crianças, questões sociodemográficas, histórico de saúde e informações relacionados às habilidades auditivas temporais, sendo os itens:
  1. Identificação.
  2. Por que você procurou atendimento fonoaudiológico para seu (sua) filho (a)? () o médico solicitou; () a escola solicitou;() percebi alteração(ões) fonoaudiológica(s); () outros
  3. Seu/sua filho(a) realiza algum tratamento? () sim () não
  4. Como você classifica a saúde do seu/sua filho(a)? () Boa () Nem ruim, nem boa () Ruim
  5. Você acha que seu/sua filho(a) escuta bem? () sim () não
  6. Como você classifica a audição do seu/sua filho(a)? () Boa () Ruim
  7. Seu/sua filho(a) apresenta dificuldade em alguma das questões abaixo?
  - Prestar atenção por um período maior de tempo. () sim () não
  - Conversar em ambientes ruidosos. () sim () não
  - Conversar com várias pessoas ao mesmo tempo. () sim () não
  - Atividades escolares. () sim () não
  - Seguir orientações. () sim () não
- Teste Token – versão reduzida^(^17^)^: O Token Test, versão reduzida^(^17^)^, utilizado para a triagem de aspectos cognitivos iniciais. Este protocolo foi desenvolvido com o objetivo de identificar alterações sutis na compreensão verbal, além de avaliar a integridade dos componentes cognitivos da linguagem. A aplicação baseia-se na emissão de comandos verbais, que devem ser executados pelo participante, permitindo mensurar o desenvolvimento linguístico em relação à idade e escolaridade. Para a aplicação do teste, foram utilizadas peças geométricas de diferentes cores e tamanhos, que facilitam a execução das instruções e tornam o protocolo mais acessível ao público infantil. O teste é composto por seis partes. A Parte 1 contém sete itens, enquanto as Partes 2, 3, 4 e 5 apresentam quatro itens cada. A Parte 6 é formada por 13 itens. Nas Partes 1, 3 e 5, são utilizadas todas as peças geométricas (grandes e pequenas), ao passo que, nas Partes 2, 4 e 6, são utilizadas apenas as peças grandes, com as peças pequenas mantidas cobertas durante a aplicação.
- Critério de Classificação Econômica Brasil (CCEB)^(^18^)^: elaborado pela Associação Brasileira de Empresas de Pesquisa (ABEP). Tem o objetivo de estimar o poder aquisitivo de compra das famílias, por meio de características domiciliares e presença de bens, além do grau de instrução do chefe da família. É aplicado sob a forma de entrevista e as perguntas referem-se ao número disponível de itens presentes na residência, existência de serviços públicos e grau de instrução do chefe da família. Para sua análise, cada item apresenta uma pontuação específica e a soma deles determinará a classificação correspondente em seis possíveis modalidades de classe: A, B1, B2, C1, C2, D-E. Quanto maior a quantidade de itens e o grau de instrução do chefe da família, maior é a pontuação, sendo o nível mais alto a classe A.
- Avaliação auditiva^(^19^)^: A avaliação foi realizada em sala acusticamente tratada, assegurando condições apropriadas para a aplicação dos procedimentos audiológicos. Inicialmente, realizou-se a inspeção do meato acústico externo (MAE), com o objetivo de verificar a ausência de obstruções ou condições que pudessem interferir nas etapas subsequentes da avaliação. Em seguida, procedeu-se à análise da imitância acústica, composta pela timpanometria e pela pesquisa dos reflexos acústicos contralaterais e ipsilaterais, utilizando o imitanciômetro Madsen Astera^(^2^)^ com sonda de 226 Hz. Por fim, foi realizada a avaliação audiológica convencional, composta pela audiometria tonal limiar, para determinação dos limiares auditivos, e pela logoaudiometria, com o propósito de avaliar a capacidade de detecção e reconhecimento da fala. Foram considerados resultados dentro dos padrões de normalidade aqueles que apresentaram curva timpanométrica tipo A presença de reflexos acústicos bilateralmente, limiares auditivos iguais ou melhores que 15 dB NA em ambas as orelhas e respostas compatíveis em *screening* audiométrico com técnica de varredura nas frequências de 500 a 4000 Hz, conforme critérios estabelecidos pela literatura especializada^(^19^)^.
- A habilidade de resolução temporal foi avaliada por meio do Random Gap Detection Test (RGDT)^(^20^)^, que mede a capacidade de detectar intervalos de silêncio entre dois estímulos sonoros. O teste, de natureza diótica, consiste na apresentação de pares de tons puros nas frequências de 500, 1000, 2000 e 4000 Hz, com intervalos de tempo variando aleatoriamente entre 0, 2, 5, 10, 15, 20, 25, 30 e 40 milissegundos. Os participantes foram instruídos a responder verbalmente se perceberam um ou dois sons. Foram considerados resultados dentro da normalidade aqueles com média de detecção inferior ou igual a 10 milissegundos. A aplicação foi realizada em sala acusticamente tratada, garantindo condições controladas de avaliação.
- A Escala de Letramento Funcional em Saúde para crianças foi elaborada pelas pesquisadoras com o objetivo de avaliar o letramento funcional em saúde de crianças. Para tanto, levou em consideração o conceito de letramento funcional em saúde e a faixa etária do estudo, além de ter o cuidado de considerar crianças, à partir de nove anos, que já são capazes de tomar decisões^(^4-6^)^. O instrumento é composto por oito questões, nas quais a criança escolhe uma entre três opções de resposta: *sempre*, *às vezes* ou *nunca*. Para facilitar a compreensão das alternativas, especialmente em faixas etárias mais jovens, foi utilizada uma escala visual ilustrada, conforme apresentado no Apêndice A. A aplicação foi realizada individualmente, com leitura assistida, quando necessário, respeitando o ritmo e o nível de compreensão da criança.
- A Classificação Internacional de Funcionalidade (CIF)^(^13^)^ é uma ferramenta elaborada pela Organização Mundial da Saúde (OMS) para sistematizar e descrever aspectos relacionados à funcionalidade e às condições de saúde de indivíduos. Essa classificação organiza, de maneira integrada, diferentes domínios da vida de uma pessoa diante de uma condição de saúde específica. Neste estudo foi contemplada a categoria comunicação (d350)^(^13^)^, que compreende o desempenho em conversação, iniciar, manter e finalizar uma troca de pensamentos e ideias, realizada por meio da linguagem escrita, oral, de sinais ou de outras formas de linguagem, com uma ou mais pessoas conhecidas ou estranhas, em ambientes formais ou informais^(^13^)^. Foram utilizados apenas os qualificadores 0 (nenhuma dificuldade) e 8 (dificuldade não especificada). Vale destacar que o qualificador 8 é utilizado quando há uma dificuldade, mas não é possível determinar o grau de dificuldade. Para a seleção de qualificadores foram consideradas as transcrições das entrevistas e informações do questionário da CIF respondidos pelos responsáveis^(^13^)^. Para avaliar se o Letramento em Saúde foi considerado adequado ou inadequado, realizou-se a soma dos escores atribuídos às respostas, sendo: 0 = "Nunca", 1 = "Às vezes" e 2 = "Sempre". Em seguida, utilizou-se a mediana como ponto de corte, cujo valor foi de 6,0 pontos, para classificar os participantes quanto ao nível de letramento.

### Análise de dados

Para o presente estudo foi definida como variável dependente a categoria comunicação (d350)^(^13^)^ e como variáveis independentes (sexo, escolaridade, tipo de escola, classificação econômica), variáveis sociodemográficas, letramento funcional em saúde e habilidades temporais.

Foi realizada análise descritiva, por meio da distribuição de frequência das variáveis categóricas e análise das medidas de tendência central e de dispersão das variáveis contínuas. Para as análises de associação foi utilizado o teste Qui-quadrado de Pearson, adotando-se um nível de significância de 5% (p < 0,05).

As respostas obtidas nos instrumentos foram organizadas, digitalizadas em um banco de dados específico. Para entrada, processamento e análise dos dados foi utilizado o software SPSS, versão 25.0.

## RESULTADOS

Dos 53 escolares participantes, a maior parte possuía nove anos (30,8%), com média de 10,31 anos, desvio padrão de 1,09 e mediana de 10 anos. A maioria era do sexo masculino (58,5%), estudava em escola pública (77,4%), pertencia à classe A/B (51,9%) do CCEB, não apresentou alteração na categoria comunicação - d350 (52,8%) e apresentou resultado alterado no teste RGDT (79,2%). Além disso, a maior parte cursava o 5º ano do ensino fundamental (28,3%) e apresentou resultado inadequado (62,3%) na escala Letramento Funcional em Saúde (Tabela 1).

**Tabela 1:** Características dos estudantes avaliados

	N	%
Sexo		
Feminino	22	41,5
Masculino	31	58,5
Total	53	100,0
Idade		
9 anos	16	30,2
10 anos	13	24,5
11 anos	14	26,4
12 anos	10	18,9
Total	53	100,0
Ano escolar		
3º	2	3,8
4º	13	24,5
5º	15	28,3
6º	14	26,4
7º	9	17,0
Total	53	100,0
Tipo de escola		
Pública	41	77,4
Privada	12	22,6
Total	53	100,0
CCEB		
A/B	28	52,8
C-D/E	25	47,2
Total	53	100
CIF d350		
Nenhuma dificuldade	28	52,8
Dificuldade não especificada	25	47,2
Letramento		
Adequado	20	37,7
Inadequado	33	62,3
Total	53	100
RGDT		
Adequado	11	20,8
Inadequado	42	79,2
Total	53	100,0


**Legenda:** CCEB = Classificação Econômica Brasil; CIF = Classificação Internacional de Funcionalidade; RGDT = Random Gap Detection Test

Na Escala de Letramento Funcional em Saúde, analisada por questão, a maior parte dos participantes respondeu a opção “sempre” nas questões: Q3 (49,1%), Q4 (54,7%) e Q8 (64,2%); a maior parte respondeu a opção “às vezes” nas questões: Q1 (54,7%), Q2 (39,6%), e Q7(43,4%); a maior parte respondeu a opção “nunca” na questão Q5 (41,5%) (Tabela 2).

**Tabela 2:** Análise da Escala de Letramento Funcional em saúde

	N	%
**Q1: Quando você vai ao fonoaudiólogo, médico, dentista ou outro profissional de saúde, você costuma prestar atenção nas orientações que são dadas aos seus pais?**		
Sempre	22	41,5
Às vezes	29	54,7
Nunca	2	3,8
Total	53	100,0
**Q2: Quando você vai ao médico, fonoaudiólogo, dentista ou outro profissional de saúde, você costuma perguntar/tirar dúvidas sobre sua saúde?**		
Sempre	12	22,6
Às vezes	21	39,6
Nunca	20	37,7
Total	53	100
**Q3: Você consegue entender as orientações que são dadas pelo fonoaudiólogo, médico, dentista ou outro profissional de saúde para você?**		
Sempre	26	49,1
Às vezes	18	34,0
Nunca	9	17,0
Total	53	100,0
**Q4: Você consegue entender as explicações dados pelos seus pais sobre a sua saúde?**		
Sempre	29	54,7
Às vezes	17	32,1
Nunca	7	13,2
Total	53	100,0
**Q5: Você costuma perguntar aos seus pais sobre o tratamento que está realizando com o fonoaudiólogo?**		
Sempre	14	26,2
Às vezes	17	32,1
Nunca	22	41,5
Total	53	100,0
**Q6:Você costuma perguntar aos seus pais sobre o tratamento/consulta que está realizando com o médico?**		
Sempre	17	32,1
Às vezes	18	34,0
Nunca	18	34,0
Total	53	100,0
**Q7: Você costuma perguntar aos seus pais sobre o tratamento/consulta que está realizando com outros profissionais de saúde?**		
Sempre	14	26,4
Às vezes	23	43,4
Nunca	16	30,2
Total	53	100,0
**Q8: Você costuma prestar atenção nas orientações que são dadas por fonoaudiólogos, médicos e dentistas?**		
Sempre	34	64,2
Às vezes	13	34,5
Nunca	6	11,3
Total	53	100,0


**Legenda:** Q = questão

Foi realizada análise de associação entre a categoria da CIF d350 e as variáveis selecionadas do estudo, por meio do teste Qui-quadrado de Pearson. Foi encontrado resultado com significância estatística na associação entre Letramento Funcional em Saúde (p=0,012). Verificou-se que as crianças sem queixas relacionadas à comunicação apresentaram níveis adequados de Letramento Funcional em Saúde, ao passo que aquelas com queixas comunicativas demonstraram maior frequência de dificuldades nesse aspecto (Tabela 3).

**Tabela 3:** Análise da associação entre CIFd350 e variáveis selecionadas

	**CIFd350**		**Valor-p** *
	**Dificuldade não especificada**	**Nenhuma dificuldade**	
**Variáveis**	**N(%)**	**N(%)**	
**Sexo**			0,442
Feminino	9 (36,0)	13 (46,4)	
Masculino	16 (64,0)	15 (53,6)	
Total	25 (100,0)	28 (100,0)	
**Idade**			0,948
9 anos	7 (28,7)	9 (33,3)	
10 anos	6 (24,0)	7 (25,9)	
11 anos	7 (28,0)	7 (25,9)	
12 anos	5 (20,0)	4 (14,8)	
Total	25 (100,0)	27 (100,0)	
**Ano escolar**			0,518
3	2 (8,0)	0 (0,0)	
4	5 (20,0)	8 (28,6)	
5	6 (24,0)	9 (32,1)	
6	7 (28,0)	7 (25,0)	
7	5 (20,0)	4 (14,3)	
Total	25 (100,0)	28 (100,0)	
**Tipo de escola**			0,823
Pública	19 (76,0)	22 (78,6)	
Privada	6 (24,0)	6 (21,4)	
Total	25 (100,0)	28 (100,0)	
**CCEB**			0,797
A/B	12 (50,0)	15 (53,6)	
C-D/E	12 (50,0)	13 (46,4)	
Total	24 (100,0)	28 (100,0)	
**RGDT**			0,898
Adequado	5 (20,0)	6 (21,4)	
Inadequado	20 (80,0)	22 (72,6)	
Total	25 (100,0)	28 (100,0)	
**Letramento em Saúde**			0,012*
Adequado	5 (20,0)	15 (53,5)	
Inadequado	20 (80,0)	16,4(24,5)	
Total	25 (100,0)	28 (100,0)	

Teste Qui-quadrado de Pearson

* valor de p≤0,05

**Legenda:** CCEB = Classificação Econômica Brasil; CIF = Classificação Internacional de Funcionalidade; RGDT = Random Gap Detection Test

Na análise de associação entre o componente da CIF d350 e a Escala de Letramento funcional em Saúde, por questão, foi encontrado resultado com significância estatística entre a questão Q1 (p=0,040) e Q2 (p=0,035), com tendência de quem foi classificado como sim no código da CIF responder nunca nas questões do letramento funcional. As demais questões não apresentaram resultados com significância estatística (Tabela 4).

**Tabela 4:** Análise da associação entre CIFd350 e questões da Escala de Letramento Funcional em Saúde

**Variáveis**	**CIFd350**		**Valor-p** *
	**Dificuldade não especificada**	**Nenhuma dificuldade**	
	**N(%)**	**N(%)**	
**Q1: Quando você vai ao fonoaudiólogo, médico, dentista ou outro profissional de saúde, você costuma prestar atenção nas orientações que são dadas aos seus pais?**			0,040
Sempre	7 (28,0)	15 (53,6)	
Às vezes	18 (72,0)	11 (39,3)	
Nunca	0 (0,0)	2 (7,1)	
Total	25 (100,0)	28 (100,0)	
**Q2: Quando você vai ao médico, fonoaudiólogo, dentista ou outro profissional de saúde, você costuma perguntar/tirar dúvidas sobre sua saúde?**			0,035
Sempre	4 (16,0)	8 (28,6)	
Às vezes	7 (28,0)	14 (50,0)	
Nunca	14 (56,0)	6 (21,4)	
Total	25 (100,0)	28 (100,0)	
**Q3: Você consegue entender as orientações que são dadas pelo fonoaudiólogo, médico, dentista ou outro profissional de saúde para você?**			0,659
Sempre	13 (52,0)	13 (46,4)	
Às vezes	9 (36,0)	9 (32,2)	
Nunca	3 (12,0)	6 (21,4)	
Total	25 (100,0)	28 (100,0)	
**Q4: Você consegue entender as explicações dados pelos seus pais sobre a sua saúde?**			0,504
Sempre	12 (48,0)	17 (60,7)	
Às vezes	10 (40,0)	7 (25,0)	
Nunca	3 (12,0)	4 (14,3)	
Total	25 (100,0)	28 (100,0)	
**Q5: Você costuma perguntar aos seus pais sobre o tratamento que está realizando com o fonoaudiólogo?**			0,202
Sempre	4 (16,0)	10 (35,8)	
Às vezes	8 (32,0)	9 (32,1)	
Nunca	13 (52,0)	9 (32,1)	
Total	25 (100,0)	28 (100,0)	
**Q6:Você costuma perguntar aos seus pais sobre o tratamento/consulta que está realizando com o médico?**			0,668
Sempre	7 (28,0)	10 (35,7)	
Às vezes	10 (40,0)	8 (28,6)	
Nunca	8 (32,0)	10 (35,7)	
Total	25 (100,0)	28 (100,0)	
**Q7: Você costuma perguntar aos seus pais sobre o tratamento/consulta que está realizando com outros profissionais de saúde?**			0,289
Sempre	7 (28,0)	7 (25,0)	
Às vezes	13(52,0)	10 (35,7)	
Nunca	5 (20,0)	11 (39,3)	
Total			
**Q8: Você costuma prestar atenção nas orientações que são dadas por fonoaudiólogos, médicos e dentistas?**			0,161
Sempre	13 (52,0)	21 (75,0)	
Às vezes	9 (36,0)	4 (14,3)	
Nunca	3 (12,0)	3(10,7)	
Total	25 (100,0)	28 (100,0)	

* Teste Qui-quadrado de Pearson

**Legenda:** Q = questão

## DISCUSSÃO

O presente estudo analisou a relação entre funcionalidade, habilidades temporais, letramento em saúde e aspectos sociodemográficos em escolares na faixa etária de nove a doze anos. A distribuição da amostra mostrou que a maioria das crianças era do sexo masculino, cursava o 5º ano escolar e era proveniente de escola pública.

Em relação ao CCEB a maioria era proveniente da classe econômica A/B e frequentava escola regular, tal fato pode ter contribuído para os resultados positivos no desempenho em Letramento Funcional em Saúde. Um estudo prévio aponta que o letramento funcional em saúde está relacionado às condições sociais, escolares e qualidade de vida^(^21^)^. Além disso, o nível de letramento funcional em saúde pode variar em acordo com as características da amostra, como sociodemográficas, culturais e familiares^(^22^)^.

A utilização da abordagem biopsicossocial da CIF para a caracterização dos aspectos funcionais referentes ao desempenho em conversação permite descrever possíveis fatores relacionados à funcionalidade, incapacidade e ao contexto dos participantes, que poderão influenciar na aquisição de habilidades importantes para a participação social, como o Letramento Funcional em Saúde^(^16^)^. Neste estudo a seleção de qualificadores, mediante as transcrições das entrevistas e informações do questionário da CIF, respondidos pelos responsáveis, permitiu identificar a percepção familiar quanto à habilidade de conversação das crianças analisadas.

A evidência de que a maioria das crianças avaliadas não apresentou queixas associadas a categoria de comunicação (d350) é importante no presente estudo. Especificamente, foram relatadas poucas dificuldades relacionadas a situações como conversar em ambientes ruidosos, interagir com múltiplos interlocutores simultaneamente, manter a atenção por períodos prolongados e seguir instruções verbais. Esses aspectos são diretamente relacionados às exigências funcionais da rotina escolar, familiar e social da criança.

Por outro lado, observou-se que aquelas crianças com queixas comunicativas apresentaram, de forma consistente, desempenho inferior na escala de Letramento Funcional em Saúde, o que sugere uma possível associação entre limitações funcionais na comunicação e menor capacidade de compreender e utilizar informações relacionadas à saúde. Este achado reforça a hipótese de que dificuldades no domínio comunicativo podem impactar diretamente a forma como as crianças acessam, processam e respondem a orientações em contextos clínicos, escolares e familiares. Tal informação indica a associação entre Letramento Funcional em Saúde à uma adequada habilidade de compreensão da mensagem, pois tal habilidade é necessária para analisar e compreender as informações de saúde, o que favorece um adequado Letramento Funcional em Saúde^(^16^)^.

Esses resultados estão em consonância com estudo prévio que aponta a comunicação como uma habilidade central para a promoção do letramento em saúde desde a infância^(^23^)^, considerando que compreender, interpretar e aplicar informações depende de competências linguísticas e cognitivas bem desenvolvidas. Além disso, ressalta-se que o ambiente comunicativo – como ruído, número de interlocutores e complexidade das instruções – pode ser um fator modulador importante dessas competências, especialmente entre crianças em fase de aquisição e consolidação da linguagem^(^23^)^.

Na Escala de Letramento Funcional em Saúde, aplicada na população avaliada, a maioria dos estudantes analisados apresentou resultado inadequado no Letramento Funcional em Saúde. Estudos prévios, realizados com adolescentes e adultos^(^21,22^)^, as análises dos dados revelaram que a maioria dos indivíduos apresentou escores de letramento funcional em saúde adequados em indivíduos adultos e adolescentes, com utilização de instrumentos variados.

Mais de um terço dos participantes apresentou desempenho inadequado no Letramento Funcional em Saúde, resultado que merece atenção, sobretudo por envolver uma população em fase de desenvolvimento. A literatura tem demonstrado uma associação significativa entre níveis mais elevados de escolaridade e maior competência em letramento funcional em saúde^(^5,7,21,22^)^. Estudos apontam que indivíduos tendem a apresentar menor probabilidade de apresentar dificuldades nessa habilidade, uma vez que a escolarização está diretamente relacionada à aquisição de vocabulário, à capacidade de compreensão e ao uso funcional da linguagem no cotidiano^(^7,24^)^. Considerando que a amostra deste estudo foi composta por crianças em processo de alfabetização e escolarização formal, matriculadas no ensino fundamental, é plausível supor que o desempenho identificado no letramento funcional em saúde esteja relacionado à etapa de desenvolvimento cognitivo e linguístico em que essas crianças se encontram. A capacidade de compreender, interpretar e aplicar informações relacionadas à saúde ainda está em construção, sendo influenciada tanto por fatores escolares quanto pelo ambiente familiar e social.

Na análise do teste RGDT, a maioria das crianças obteve resultados considerados alterados. Em estudo realizado com crianças com transtorno fonológico, foi constatado um baixo desempenho em todos os testes de processamento temporal utilizados nos sujeitos da pesquisa^(^25^)^. Neste estudo, a maioria das crianças apresentou queixa quanto às dificuldades escolares, o que pode estar correlacionado com o desempenho do teste RGDT. Além disso, uma possível explicação para o elevado número de alterações é o fato de pais com maior preocupação com o desenvolvimento dos escolares terem atendido ao convite realizado nas redes sociais.

Quanto à análise de associação entre o resultado do teste RGDT e a categoria comunicação (d350), não foi observado significância estatística. Sendo esta categoria respondida mediante aplicação do questionário da CIF e da anamnese com os pais/responsáveis, a maioria informou que as crianças não apresentavam dificuldades na habilidade de conversação dessa categoria. Esse resultado deve ser interpretado com cautela. Em outro estudo, participantes relataram dificuldade para se comunicar em contextos familiares, como conversar em casa ou seguir instruções em ambientes com múltiplos estímulos^(^15^)^. Além disso, as crianças relataram conversar pouco com seus pais, sendo reduzidos os momentos de conversação no ambiente familiar^(^15^)^. Tais discrepâncias podem estar associadas a diferentes fontes de informação (autorrelato da criança versus relato do responsável), à subjetividade da percepção parental ou a características contextuais que influenciam a manifestação das dificuldades comunicativas em situações específicas. Sendo assim, são necessárias outras investigações desta habilidade, com avaliações específicas dos estudantes em estudos posteriores e maior esclarecimento em relação à dinâmica familiar.

A possível relação entre os aspectos temporais e a categoria comunicação (d350), possibilita identificar limitações nas tarefas de recepção e a expressão da linguagem oral, o que pode influenciar nas atividades de participação social. Além desses fatores, é necessário considerar os fatores pessoais e ambientais, dentre eles a idade escolar e as dificuldades escolares para compreensão das particularidades de cada indivíduo^(^26,27^)^. Desta forma, o uso dos qualificadores em associação a habilidades pessoais, favorece a visualização das limitações de atividades e restrições de participação que podem ser ocasionadas por alterações das funções auditivas associadas a fatores ambientais, sendo que tais alterações poderão influenciar na qualidade de vida das pessoas^(^27,28^)^.

Como limitações do presente estudo vale destacar o tamanho da amostra, bem como sua seleção (não probabilística), fatores que não permitem a generalização dos resultados. Além disso, apesar da ampla divulgação para recrutamento com uso de mídias sociais, a adesão foi baixa para a pesquisa. Ainda em relação à amostra, vale ressaltar que o número reduzido de participantes de escolas de financiamento privado compromete a análise da variável tipo de escola.

Há avanços que merecem ser considerados, tais como a triangulação entre modelo biopsicossocial, a resolução temporal e o letramento em saúde. Além disso, é um estudo inovador ao abordar o letramento em saúde e processamento auditivo de escolares, que leva em consideração o modelo biopsicossocial de saúde preconizado pela OMS, que integra aspectos biológicos, psicológicos e sociais na compreensão do indivíduo. A proposta metodológica contribui para aproximar a prática clínica das diretrizes internacionais de avaliação centrada na funcionalidade, promovendo uma visão mais ampla e contextualizada do desenvolvimento infantil. Do ponto de vista prático, os achados reforçam a importância de se considerar o letramento em saúde desde a infância, especialmente em populações em fase de aquisição da linguagem e em contextos escolares. A detecção de dificuldades comunicativas e de compreensão de informações relacionadas à saúde pode favorecer intervenções interdisciplinares mais eficazes, com efeitos positivos no desempenho acadêmico, na adesão a orientações clínicas e no desenvolvimento da autonomia comunicativa.

Para estudos futuros, recomenda-se a ampliação da amostra, a inclusão de múltiplos informantes (criança, pais e professores) e a aplicação de instrumentos padronizados e validados para diferentes contextos linguísticos e culturais. A continuidade de investigações que articulem aspectos funcionais, habilidades auditivas e letramento em saúde poderá contribuir para o delineamento de estratégias educativas e preventivas mais efetivas, com foco na promoção da saúde infantil e no fortalecimento da comunicação como determinante do cuidado.

## CONCLUSÃO

Os resultados revelaram associação entre a categoria comunicação d350 dos escolares e o Letramento Funcional em Saúde. Norteada pelos princípios da atenção integral e promoção da saúde, e utilizando a base conceitual da CIF, pode-se associar o desempenho em Letramento Funcional em Saúde e as habilidades de conversação com os aspectos de Atividades, Participação e Fatores Ambientais das crianças.

Assim, foi possível perceber que o Letramento Funcional em Saúde é composto por estruturas sociais complexas e multifatoriais. Dessa forma, reforça-se a importância de novos estudos que permitam a associação entre as habilidades pessoais, ambientais e sociais para melhor compreensão do Letramento Funcional em Saúde em crianças.

## Apêndice A Escala Letramento Funcional em saúde

**Table:**

**LETRAMENTO EM SAÚDE EM CRIANÇAS E ADOLESCENTES**												
**1.**	Quando você vai ao fonoaudiólogo, médico, dentista ou outro profissional de saúde, você costuma prestar atenção nas orientações que são dadas aos seus pais?											
		
	Sempre			Às vezes				Nunca				
**2.**	Quando você vai ao médico, fonoaudiólogo, dentista ou outro profissional de saúde, você costuma perguntar/tirar dúvidas sobre sua saúde?											
		
	Sempre			Às vezes					Nunca			
**3.**	Você consegue entender as orientações que são dadas pelo fonoaudiólogo, médico, dentista ou outro profissional de saúde para você?											
		
	Sempre					Às vezes				Nunca		
**4.**	Você consegue entender as explicações dados pelos seus pais sobre a sua saúde?											
		
	Sempre					Às vezes					Nunca	
**5.**	Você costuma perguntar aos seus pais sobre o tratamento que está realizando com o fonoaudiólogo?											
		
	Sempre		Às vezes									Nunca
**6.**	Você costuma perguntar aos seus pais sobre o tratamento/consulta que está realizando com o médico?											
		
	Sempre			Às vezes								Nunca
**7.**	Você costuma perguntar aos seus pais sobre o tratamento/consulta que está realizando com outros profissionais de saúde?											
		
	Sempre				Às vezes			Nunca				
**8.**	Você costuma prestar atenção nas orientações que são dadas por fonoaudiólogos, médicos e dentistas?											
		
	Sempre	Às vezes					Nunca
